# Study on the Synthesis and Electrochemical Properties of Nitrogen-Doped Graphene Quantum Dots

**DOI:** 10.3390/ma17246163

**Published:** 2024-12-17

**Authors:** Yongbo Wang, Yanxiang Wang, Dongming Liu, Yanqiu Feng, Deli Yang, Simeng Wu, Haotian Jiang, Donglong Wang, Shishuai Bi

**Affiliations:** 1 Key Laboratory for Liquid-Solid Structural Evolution and Processing of Materials, State Key Laboratory of Crystal Materials, Shandong University, Jinan 250061, China; wyongbo1011@163.com (Y.W.);; 2 Carbon Fiber Engineering Research Center, School of Materials Science and Engineering, Shandong University, Jinan 250061, China; 3 Shandong Jinhong New Material Co. Ltd., Weifang 262100, China

**Keywords:** N-GQDs, hydrothermal method, electrochemical characterization, energy storage

## Abstract

Nitrogen-doped graphene quantum dots (N-GQDs) are widely used in biosensing, catalysis, and energy storage due to their excellent conductivity, high specific surface area, unique quantum size effects, and optical properties. In this paper, we successfully synthesized N-GQDs using a facile hydrothermal approach and investigated the effects of different hydrothermal temperatures and times on the morphology and structure of N-GQDs. The results indicated that the size of N-GQDs gradually increased and they eventually aggregated into graphene fragments with increasing temperature or reaction time. Notably, N-GQDs synthesized at 180 °C for 6 h exhibited the most uniform size, with an average diameter of approximately 3.48 nm, a height of 5–6 graphene layers, as well as favorable fluorescence properties. Moreover, the surface of N-GQDs contained abundant oxygen- and nitrogen-containing functional groups, which could provide numerous active sites for electrode reactions. The assembled electrode exhibited typical pseudocapacitive behavior with exceptional electrochemical performance, achieving a specific capacitance of 102 F g^−1^ at a current density of 1 A g^−1^. In a 10,000-cycle test, the electrode demonstrated excellent cycling stability with a capacitance retention rate of 78.5%, which laid the foundation for practical application of the electrode. This work successfully applied N-GQDs in supercapacitors, offering new insights into their development for the energy storage field.

## 1. Introduction

Graphene quantum dots (GQDs), as a novel type of graphene-based nanomaterial with sizes typically below 10 nm, exhibit remarkable electrical conductivity, high specific surface area, unique optical properties, and biocompatibility, making GQDs widely applicable in photocatalysis, bioimaging, sensing, and energy storage [1,2,3,4]. There are currently two main methods for preparing GQDs: the “top–down” method and the “bottom–up” method [5,6]. The “top–down” method involves cutting large-sized carbon materials (carbon fibers [7,8], graphene [9]) into small-sized quantum dots. Although this method has the advantages of abundant raw materials, controllable thickness, and suitability for large-scale production, the resulting GQDs suffer from uncontrollable size and structural defects due to the use of strong acids or oxidants during processing [10]. By contrast, the “bottom–up” method involves polymerizing and carbonizing small molecule compounds to generate GQDs, which have advantages such as controllable size and shape, stable structure, and controllable surface functional groups [11]. The “bottom–up” methods mainly include the hydrothermal method [12], solvothermal method [13], and pyrolysis method [14]. Due to its diverse precursors, ease of doping with heteroatoms, and controllable size and shape, the hydrothermal method is widely used in the preparation of GQDs [15].

In order to improve the optical, electrical, and chemical properties of GQDs and facilitate their application in catalysis, biological imaging, and energy storage fields, researchers typically introduce heteroatoms into GQDs [16,17]. Common heteroatoms include nitrogen [18], sulfur [19,20], and phosphorus [21], and research on N-GQDs is the most extensive. Although nitrogen atoms have a similar atomic radius to carbon atoms, they have higher electronegativity. Therefore, when nitrogen atoms enter the carbon skeleton of GQDs, they can provide additional electrons and improve the electron cloud density, enhancing the conductivity and electron transport ability of the material [22]. Nitrogen atoms can also alter the band structure of GQDs and regulate their band gap, which can improve the fluorescence emission characteristics of GQDs [23]. In addition, nitrogen doping usually introduces functional groups such as amino groups and pyrrole nitrogen, which can enhance the hydrophilicity and surface activity of GQDs. However, current methods for preparing N-GQDs often suffer from low yield, size inconsistency, and poor fluorescence stability. Therefore, the preparation of structurally stable and uniformly sized N-GQDs has become a current research focus.

Due to the excellent conductivity, high specific surface area, and abundant functional groups on the surface of N-GQDs, their application in energy storage has attracted much attention [24]. Supercapacitors have become increasingly appealing due to their high-power density, fast charging and discharging, wide operating temperature, and long cycle life [25,26,27,28,29]. At present, the application of N-GQDs in supercapacitors mainly focuses on the electrochemical research preparing composite electrodes with pseudocapacitive materials (conductive polymers [30], transition metal oxides [31]), while there is relatively little research on the capacitance behavior of N-GQDs. However, nitrogen doping can alter the electronic structure of GQDs, leading to faster electron or ion transfer at the interface between the material and electrolyte, which endows GQDs with superior electrochemical response. Therefore, exploring the electrochemical behavior of N-GQDs will provide insight into how nitrogen atoms influence the structure of GQDs, their charge storage mechanisms, and the catalytic activity of different nitrogen configurations. This will help to promote the application of N-GQDs in energy storage and catalysis fields.

This paper systematically investigates the effects of different hydrothermal temperatures and times on the morphology and structure of N-GQDs using a simple hydrothermal method and analyzes the functional group distribution on the surface of N-GQDs using Fourier-transform infrared spectroscopy and X-ray photoelectron spectroscopy methods. Finally, a three-electrode system is used for electrochemical testing. The results showed that the average size of N-GQDs prepared at 180 °C for 6 h was about 3.48 nm, with the most uniform distribution and good fluorescence characteristics. In addition, the surface of N-GQDs contained a large number of oxygen-containing or nitrogen-containing functional groups, which enhanced their surface activity and offered abundant active sites for electrode reactions. In electrochemical testing, N-GQDs-2 exhibited excellent electrochemical performance, with a specific capacitance of 102 F g^−1^ at 1 A g^−1^ and a capacitance retention rate of 78.9% after 10,000 cycles, demonstrating excellent cycling performance.

## 2. Experimental Section

### 2.1. Materials

All reagents used in this experiment were analytical grade without further purification. Citric acid (CA), ethylenediamine (EDA), anhydrous ethanol, potassium hydroxide (KOH), and concentrated hydrochloric acid (12 M) were purchased from McLean Co., Ltd. (Shanghai, China). Active carbon, conductive carbon black, and polytetrafluoroethylene suspension (PTFE, 60 wt%) were purchased from Yamei Nano Co., Ltd. (Jiaxing, China). The dialysis bag (1000 Da) and 0.22 um filter head were purchased from Bickman Biotechnology Company (Changsha, China). Foam nickel (thickness: 1 mm) was purchased from Guangjiayuan New Material Co., Ltd (Kunshan, China). Deionized water was produced by the ultrapure water machine.

### 2.2. Preparation of N-GQDs

In this paper, the “bottom–up” method was used to prepare N-GQDs. Firstly, 3 g CA and 1 g EDA were dissolved in 30 mL deionized water and transferred to a reaction vessel for hydrothermal reaction at 180 °C for 6 h. Then, the orange red solution was filtered through a 0.22 μm filter head and dialyzed in a 1000 Da dialysis bag for 2 days. Finally, the pale yellow solution was frozen for 3 days to obtain N-GQD powder.

In order to investigate the effects of temperature and time on the morphology and structure of quantum dots, the hydrothermal parameters were set to 160 °C -6 h, 180 °C -6 h, 200 °C -6 h, 180 °C -4 h, 180 °C -8 h, and 180 °C -12 h, respectively, and named N-GQDs-X, where X is equal to 1, 2, 3, 4, 5, and 6.

### 2.3. Preparation of N-GQD Composite Electrode

Firstly, foam nickel was washed in ethanol, 0.5 M hydrochloric acid, and deionized water to remove surface oil and oxide, then dried at 60 °C for 8 h, and finally cut into 1 × 1 cm^2^ current collector. N-GQDs, conductive carbon black, and polytetrafluoroethylene suspension (5 wt%) were dissolved in an appropriate amount of alcohol in a mass ratio of 8:1:1 and sonicated for 15 min. The suspension was then applied to foam nickel and dried at 60 °C for 12 h. Finally, the composite electrode was pressed into electrode sheets on the tablet press at 6–8 Mpa. The mass of N-GQDs coated on the electrode sheet was approximately 2 mg, and the mass ratio of Ni to N-GQDs in the electrode was 25:1.

### 2.4. Material Characterization

Cold field emission transmission electron microscopy (JEM F200, JEOL Ltd., Akishima, Tokyo, Japan) and atomic force microscopy (AFM, BioScope Resolve, Bruker Ltd., Santa Barbara, California, US) were used to characterize the surface structure and 3D morphology of N-GQDs, respectively. The phase structure and surface defects of N-GQDs were analyzed using X-ray diffraction (XRD, DMAX-2500PC, Rigaku Corp., Tokyo, Japan) and Raman spectroscopy (PHS-3C), respectively. The radiation source of XRD was Cu K α radiation (λ = 0.154 nm), while the laser for Raman spectroscopy was 533 nm with a wavelength range of 1000–2000 cm^−1^. Fourier-transform infrared spectroscopy (FTIR, Tensor II, Bruker Optik GmbH, Ettlingen, Germany) and X-ray photoelectron spectroscopy (XPS, AXIS SUPRA, Shimadzu, Kyoto, Japan) were used to analyze the surface functional groups and valence electron states of N-GQDs, respectively. The ultraviolet–visible UV-Vis) spectra and Zeta potentials were obtained using a UV spectrophotometer (UV-3600, Shimadzu, Kyoto, Japan) and a Zeta potential analyzer (Nano ZS, HORIBA, Kyoto, Japan), respectively.

### 2.5. Electrochemical Testing

The N-GQD composite electrode was tested using a three-electrode system with an electrochemical workstation (Chi760e, Chenhua, Shanghai), where the N-GQD electrode was used as the working electrode, and the saturated calomel electrode and platinum electrode were used as the reference electrode and counter electrode, respectively. The electrolyte was 6 M KOH. The voltage window for CV testing was −0.6 V–0.6 V, with a scanning speed of 10 mv s^−1^–100 mv s^−1^. The current density of GCD was set to 1 A g^−1^–1.8 A g^−1^. The frequency range of EIS was 10^−2^–10^5^ Hz with an amplitude of 5 mV.

For a two-electrode system, N-GQDs-2 and activated carbon were used as the positive and negative electrodes, respectively, to form an asymmetric supercapacitor (ASC), with 6 M KOH as the electrolyte. The mass ratio of N-GQDs to activated carbon was determined according to Formula (1):

m_+_/m_−_ = C_−_ × ∆V_−_/C_+_ × ∆V_+_ (1)

where m, C, and ∆V are the mass of the active material, specific capacitance, and voltage window, respectively. For activated carbon electrodes, the specific capacitance was 150 F g^−1^ and the voltage window was −1 V–0 V.

The specific capacitance was calculated according to Formula (2):

(2) $$C_{a}=\frac{I\times ∆t}{m\times ∆V}$$

where C_a_ is the specific capacitance of the electrode, I (A) is the current, ∆t (s) is the discharge time of the electrode, m (g) is the mass of the active material, and ∆V (V) is the voltage window.

In order to analyze the pseudocapacitive behavior of the electrode materials in electrochemical reactions, we fitted the pseudocapacitance. The behavior of pseudocapacitance is usually fitted by the relationship between scan speed and current. The common fitting formula is expressed as follows, which describes the relationship between the current (i) and scan rate (v) in b-value analysis:

i = av^b^ (3)

where a and b are fitting parameters. When b = 0.5, it indicates that the capacitance behavior is diffusion controlled, while when b = 1, it indicates that the capacitance is pseudocapacitance controlled by the surface.

In addition, the current response can be further decomposed into the contributions of pseudocapacitance and double-layer capacitance, using the following formula:

I(V) = k_1_·v + k_2_·v^0.5^ (4)

where I (A) is the current at different potentials, v (V s^−1^) is the scanning speed, and k_1_ and k_2_ are constants. Among them, k_1_·v represents the contribution of pseudocapacitance, and k_2_·v^0.5^ represents the diffusion-controlled current. The contribution rate of pseudocapacitance to capacitance can be obtained by calculating k_1_·v.

## 3. Results and Discussion

**Figure 1:**
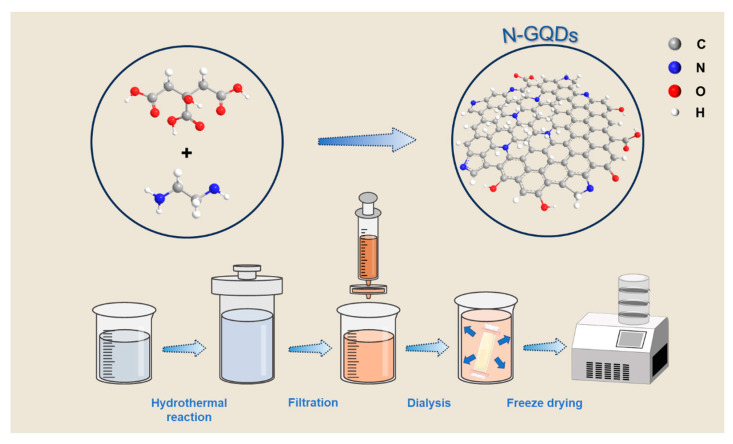
Process diagram of N-GQDs.

The process diagram for synthesizing N-GQDs is shown in Figure 1. Firstly, citric acid molecules undergo decarboxylation and dehydration reactions to produce intermediates such as acrylic acid or maleic acid under high-temperature hydrothermal conditions. At the same time, ethylenediamine reacts with these organic molecules to form nitrogen-containing small molecules. As the hydrothermal process progresses, small molecules undergo further condensation reactions and gradually form a carbon core structure, and nitrogen atoms will be incorporated into the carbon core skeleton, forming a nitrogen-doped carbon skeleton. Finally, carbon atoms form sp^2^-hybridized graphene structures through conjugation at high temperatures. In hydrothermal reactions, the surface of N-GQDs will carry a certain amount of oxygen-containing or nitrogen-containing functional groups, which can provide pseudocapacitance for subsequent electrode reactions.

**Figure 2:**
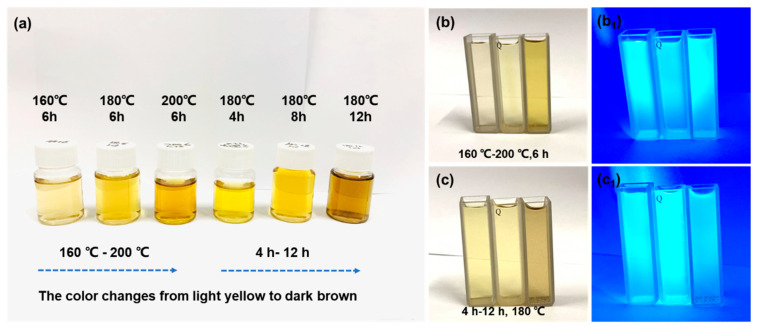
(**a**) N-GQD solutions under different hydrothermal conditions; (**b**,**b_1_**) N-GQD solutions at different temperatures under normal light and 365 nm ultraviolet lamp; (**c**,**c_1_**) N-GQD solutions at different times under normal light and 365 nm ultraviolet lamp.

**Figure 3:**
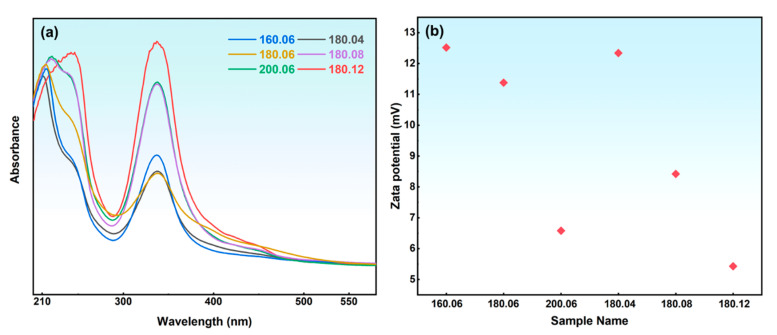
(**a**) UV spectra of N-GQDs; (**b**) Zeta potentials of N-GQDs.

Different hydrothermal conditions can affect the size and structure of N-GQDs, resulting in different fluorescence characteristics. Figure 2 shows an optical photograph of the N-GQD solution, with a concentration of approximately 0.2 mg mL^−1^. The color of the N-GQD solution gradually deepens with an increase in hydrothermal temperature or time. In addition, when the temperature reaches 200 °C or the time exceeds 12 h, there are visible black precipitates in the solution, which may be large-sized graphene fragments. This indicates that N-GQDs can further grow, aggregate, and ultimately become graphene fragments at higher temperatures or times. Due to the quantum confinement effect, the size of N-GQDs can affect their bandgap, which in turn influences the fluorescence properties of N-GQDs. Figure 2b,c show the fluorescence images of N-GQDs under a 365 nm ultraviolet lamp at different temperatures and times. All samples exhibit good fluorescence characteristics, and the fluorescence color of the N-GQD solution gradually changes from blue to green or orange with increasing temperature or time. This is attributed to the narrowing of the bandgap of large-sized N-GQDs, which exhibit a certain red shift. The size-dependent fluorescence properties of N-GQDs make them widely used in biological imaging and biological probes. Figure 3a shows the UV spectra of N-GQDs; all samples exhibit two distinct characteristic peaks at 210 nm and 340 nm, respectively. The peak at 210 nm corresponds to the π–π* transition of C=C, while the characteristic peak at 340 nm corresponds to the n–π* transition of C=O or N-H [32]. As the hydrothermal temperature or time increases, the UV spectra of N-GQDs undergo varying degrees of red shift, which is closely related to the size of N-GQDs. It is generally believed that an increase in size will reduce the bandgap width and lead to an increase in absorption wavelength. In addition, the 340 nm characteristic peak reflects the surface functional groups or nitrogen doping degree of N-GQDs. In order to analyze the dispersion stability of N-GQDs in aqueous solution, Zeta potential analysis was performed, as shown in Figure 3b. Different synthesis conditions significantly affect the surface charge and stability of N-GQDs. As the temperature or time increases, the Zeta potential gradually decreases, indicating that the electrostatic repulsion of N-GQDs in the solution gradually decreases and the stability of the solution deteriorates. In addition, the Zeta potential of N-GQDs-2 is relatively high, indicating that its surface may have more functional groups, which increases the electrostatic repulsion of quantum dots and enhances the dispersion stability of the solution.

**Figure 4:**
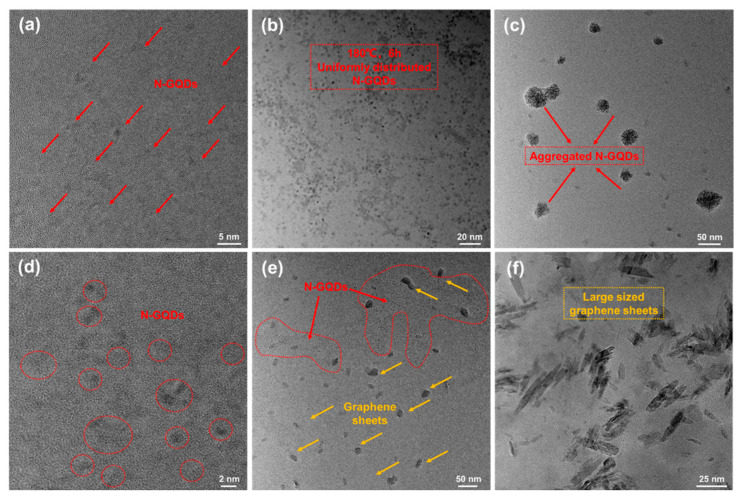
TEM images of N-GQDs. (**a**) N-GQDs-1; (**b**) N-GQDs-2; (**c**) N-GQDs-3; (**d**) N-GQDs-4; (**e**) N-GQDs-5; (**f**) N-GQDs-6.

The TEM images (Figure 4) show the surface morphology and structure of N-GQDs. As shown in the figure, when the hydrothermal temperature is 160 °C or the time is 4 h, the shape of N-GQDs is mostly circular or elliptical, and the size is relatively small, about 1–2 nm. As the temperature or time increases, the size of quantum dots also gradually increases, and their shape changes from elliptical to “long strip” shaped. When the hydrothermal conditions are 180 °C for 6 h, N-GQDs-2 exhibit good dispersion and size uniformity, with an average size (Table S1) of approximately 3.48 nm. However, as the temperature or time continues to increase, quantum dots begin to undergo further growth and aggregation (Figure 4e). Eventually, N-GQDs transform into larger graphene fragments (Figure 4f). It can be seen that hydrothermal temperature and time are key factors in regulating the size of N-GQDs. When the temperature or time is low, N-GQDs begin to “grow” and their size and yield are relatively small. When the temperature or time further increases, N-GQDs undergo carbonization growth, forming uniformly sized N-GQDs. However, when the temperature or time is too long, N-GQDs will agglomerate, forming large-sized graphene sheets, which significantly reduces the specific surface area and lowers their surface activity. Figure S1 shows the AFM image of N-GQDs. It can be seen from the figure that the height of N-GQDs-2 is about 1.88 nm, which is about 5–6 layers of graphene. Thinner quantum dots have larger band gaps and stronger quantum confinement effects, which are advantageous for their unique optical and electronic properties. In addition, the height variation is consistent with the size variation. From the above results, it can be concluded that N-GQDs-2 has good size distribution, abundant surface functional groups, and solution stability.

**Figure 5:**
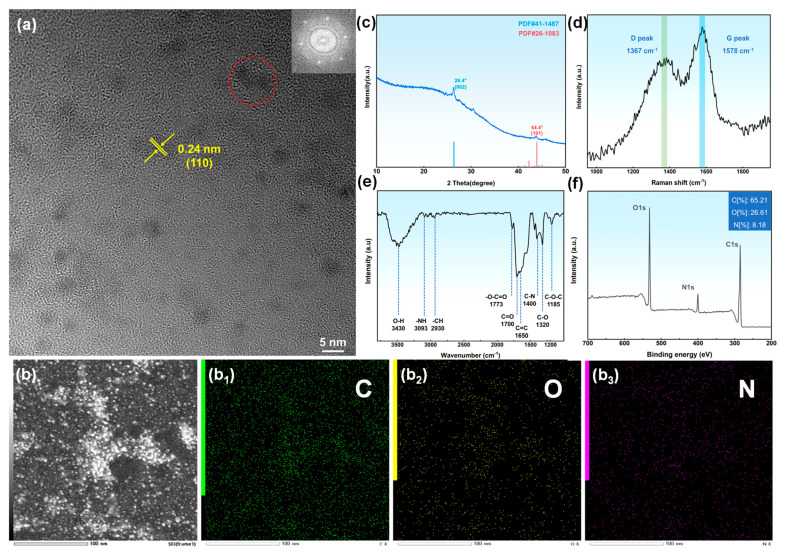
(**a**) TEM image of N-GQDs-2, with the FFT image of the red area in the upper right corner; (**b**–**b_3_**) EDS images of N-GQDs-2; (**c**) XRD pattern of N-GQDs-2; (**d**) Raman spectrum of N-GQDs-2; (**e**) infrared spectrum of N-GQDs-2; (**f**) XPS spectrum of N-GQDs-2.

In order to further investigate the structure and surface functional groups of N-GQDs, a series of characterizations were carried out on N-GQDs-2, as shown in Figure 5. Figure 5a shows the TEM image of N-GQDs-2, indicating uniform distribution with a size of approximately 2–4 nm. In addition, the lattice stripes of N-GQDs are shown in the figure, with an interplanar spacing of approximately 0.24 nm, which corresponds to the (110) plane of graphene [33]. The image in the upper right corner is the FFT image of the red area with a regular hexagonal shape, which is a typical feature of graphene structure. The EDS image of N-GQDs-2 (Figure 5b) indicates that nitrogen atoms were successfully doped into the carbon skeleton during the carbonization process. The XRD pattern of N-GQDs shows two characteristic peaks located at 26.4° and 44.4°, corresponding to the (002) and (101) crystal planes of graphite, respectively. This once again indicates the graphite structure of N-GQDs, which is consistent with the TEM results. In addition, the Raman spectrum of N-GQDs exhibits two characteristic peaks at 1367 cm^−1^ and 1578 cm^−1^, respectively, which are typical Raman structures of carbon materials. The D peak at 1367 cm^−1^ is related to sp^3^-hybridized carbon atoms, corresponding to disordered structures such as defects, while the G peak at 1578 cm^−1^ is related to the planar C=C bond vibration of sp^2^-hybridized carbon atoms, corresponding to the ordered graphite structure of the material. The D peak intensity of N-GQDs-2 is comparable to the G peak intensity, indicating that there are many defects, which may be caused by structural changes due to nitrogen atom doping. Figure 5e shows the infrared spectrum of N-GQDs, which indicates that the surface of N-GQDs contains abundant oxygen-containing and nitrogen-containing functional groups. The characteristic peak at 1650 cm^−1^ corresponds to the stretching vibration of C=C in the aromatic ring, which reflects the sp^2^-hybridized carbon structure. The absorption peaks at 3430 cm^−1^, 1773 cm^−1^, 1700 cm^−1^, 1320 cm^−1^, and 1185 cm^−1^ correspond to O-H, -O-C=O, C=O, C-N, and C-O-C groups, respectively, indicating that the surface of N-GQDs contains abundant functional groups. In addition, the absorption peaks at 3093 cm^−1^ and 1400 cm^−1^ correspond to the -NH and C-N groups, respectively, and the introduction of C-N bonds may enhance the electron transfer ability and catalytic activity of the material [34]. The XPS full spectrum of N-GQDs (Figure 5f) shows three characteristic peaks of C (~284 eV), O 1s (~532 eV), and N 1s (~400 eV), with contents of 65.21%, 26.61%, and 8.18%, respectively. This indicates that the surface of N-GQDs contains a large number of oxygen-containing and nitrogen-containing functional groups, which can improve the hydrophilicity and chemical reactivity of the material. Figure S2 shows the high-resolution XPS spectra of C 1s, O 1s, and N 1s. C1s can be fitted into four peaks: 284.65 eV (C=C), 286.05 eV (C-O/C-N), 288.4 eV (C=O), and 289.9 eV (O-C=O). The C 1s spectrum indicates the presence of numerous sp² carbon structures and a small number of oxygen- and nitrogen-doped sites in N-GQDs. O 1s can be divided into two peaks at 532.2 eV (C-O) and 531.6 eV (C-O), indicating that the surface of N-GQDs contains abundant C-O and C=O groups, which can improve the water solubility and reactivity of material. In addition, C=O can enhance the pseudocapacitive properties of the material [35]. The N 1s spectrum indicates that nitrogen in N-GQDs mainly exists in the form of graphitic nitrogen (400.46 eV) and pyrrolic nitrogen (399.96 eV). Although graphite nitrogen does not directly participate in redox reactions, it can enhance the conductivity of the material, which can promote the rapid transfer of charges and ions in the electrode. In addition, pyrrole nitrogen can participate in redox reactions to provide pseudocapacitance for the electrode [36,37]. From the above analysis, it can be concluded that the surface of N-GQDs-2 contains abundant functional groups, which can increase the surface activity of the material and provide abundant reaction sites for the pseudocapacitive properties of the material.

**Figure 6:**
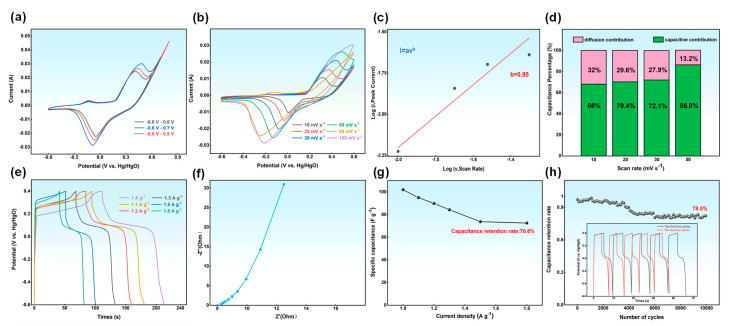
(**a**) CV curves of the N-GQDs-2 electrode at different voltage windows; (**b**) CV curves of N-GQDs-2 electrode; (**c**) linear fitting line between current and voltage; (**d**) the contribution ratios of pseudocapacitance of N-GQDs-2 at different scanning speeds; (**e**) GCD curves of N-GQDs-2; (**f**) EIS curve of N-GQD-2; (**g**) specific capacitance of N-GQDs-2; (**h**) cycling curve of N-GQDs-2.

Figure S3 shows the CV curve of N-GQDs-X. It can be seen from the figure that all N-GQDs-X exhibit redox peaks, indicating their excellent pseudocapacitive characteristics. For N-GQDs-2, its CV area is the largest, corresponding to the best electrochemical performance. In order to analyze the electrochemical performance of the N-GQDs-2 electrode, a three-electrode system was used for electrochemical testing with 6M KOH as the electrolyte. Figure 6a shows the CV curves of the N-GQDs-2 electrode under different voltage windows. When the voltage window is −0.6 V–0.6 V, the CV curve exhibits regular redox peaks. However, when the voltage exceeds 0.6 V, the CV curve shows a sharp peak, which is attributed to the oxidation reaction of carboxyl groups under alkaline conditions. However, due to the poor reversibility of this oxidation reaction, the pseudocapacitance provided by the carboxyl groups is limited. To ensure the long-term cycling stability of the material, the voltage window of N-GQDs-2 was selected as −0.6 V–0.6 V. The CV curve of N-GQDs-2 (Figure 6b) indicates that the capacitance behavior of the electrode material is contributed by both double-layer capacitance and pseudocapacitance. Due to the presence of a large number of oxygen-containing or nitrogen-containing functional groups on the surface of the material, multiple oxidation–reduction peaks appear on the CV curve, among which the oxidation peak at 0.3 V–0.5 V corresponds to the oxidation reaction of pyrrolic nitrogen or hydroxyl [38]:

C-NH + OH^−^ →C = N + H_2_O + e^−^ (5)

-OH → = O + H^+^ + e^−^ (6)

The reduction peak at −0.4 V–0.2V corresponds to the reduction reaction of carbonyl and pyrrolic nitrogen. In addition, the oxidation peak near 0 V may be related to the oxidation of pyridinic nitrogen or amino groups. As the scanning speed further increases, the oxidation–reduction peak is destroyed due to the polarization effect of the double layer on the electrode surface and the limitation of the charge transfer rate. In order to further analyze the capacitance behavior of the N-GQDs-2 electrode, the fitting analysis of current was conducted. Figure 6c shows the fitting result based on Formula (3), where b = 0.95, indicating that the capacitance behavior of the electrode material is mainly pseudocapacitance. To determine the contribution rate of pseudocapacitance to capacitance, Formula (4) was used to further fit the current, and the results are shown in Figure 6d. The capacitor is composed of double-layer capacitors and pseudocapacitors at lower scan speeds. However, as the scan speed increases, the concentration gradient of the reactants on the electrode surface increases, resulting in more reactants participating in redox reactions. Therefore, the contribution rate of pseudocapacitors gradually increases. When the scan speed is 50 mv s^−1^, the contribution rate of pseudocapacitors reaches 86.8%, which can improve the energy density of the electrode and enhance its practical application ability.

Figure 6e shows the GCD curve of N-GQDs-2. As shown in the figure, the CGD curve exhibits obvious pseudocapacitive characteristics, indicating that charge storage mainly relies on rapid surface redox reactions. The charging and discharging time are significantly shortened with the increase in current density. This is because the electrons or ions in the electrode material can complete the transfer in a shorter time at high current density. In addition, the internal resistance (IR drop) of the electrode also increases with the increase in current density, which can lead to a decrease in the charging and discharging efficiency of the electrode. The specific capacitance at different current densities was calculated according to Formula (2), as shown in Figure 6g. The specific capacitance of N-GQDs-2 is 102 F g^−1^ and it has good rate performance. In order to analyze the internal resistance of N-GQDs-2, EIS analysis was conducted. The EIS curve (Figure 6f) is composed of a high-frequency semicircle and a low-frequency straight line, where the radius of the semicircle is the charge transfer resistance R_ct_, the intercept of the semicircle is the equivalent series R_s_, and the straight line in the low-frequency region is related to the double layer or pseudocapacitance. As shown in the figure, the R_ct_ and R_s_ values of the electrode are relatively small, indicating good transport behavior between the electrode and electrolyte interface, which can promote rapid transfer of ions or charges and accelerate energy storage efficiency. The nearly vertical straight line in the low-frequency region represents the high pseudocapacitive characteristics of the electrode. In order to further evaluate the practical applicability of the N-GQDs-2 electrode, cyclic stability testing was performed (Figure 6h). In the early stage of cycling, the loss of active sites caused by partially irreversible oxidation–reduction reactions, interface impedance between electrodes and electrolytes, and shrinkage or expansion of the material’s microstructures leads to a decrease in cycling performance. But as the number of cycles increases, the structure and interface of the material gradually stabilize. After 10,000 cycles, the capacitance retention rate of the electrode is still as high as 78.5%, indicating its excellent cycling stability.

**Figure 7:**
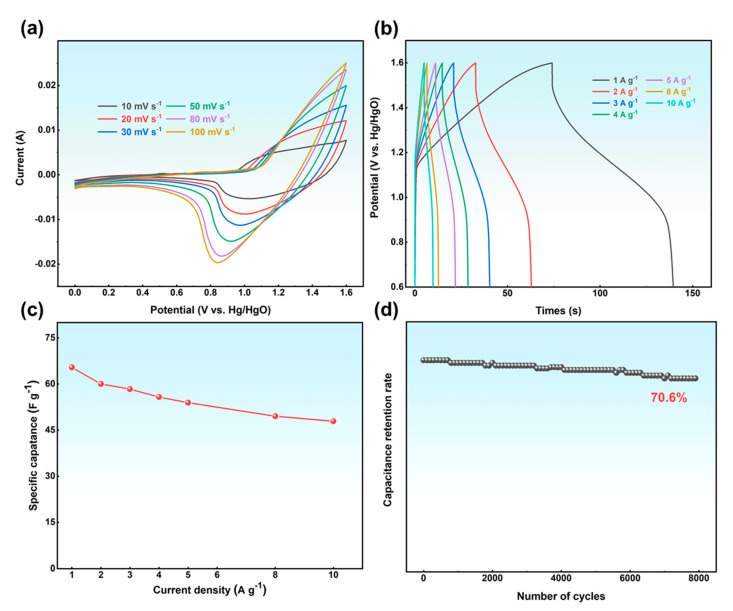
(**a**) CV curves of ACS; (**b**) GCD curves of ASC; (**c**) specific capacitance of ASC; (**d**) cycling curve of ASC.

To evaluate the practical application performance of the N-GQDs-2 electrode, it was assembled with activated carbon electrode to form an asymmetric supercapacitor (ASC). Figure 7a shows the CV curve of ASC with a voltage window of 0 V–1.6V. As the scanning speed increases, the oxidation peak and reduction peak move toward positive and negative potentials, respectively, but maintain a clear peak shape, indicating that ASC has good rate performance. Figure 7b shows the GCD curve of ASC, which appears as an asymmetric triangle—this is a typical feature of pseudocapacitive materials. The vertical voltage drop at the top of the curve corresponds to the internal resistance of ASC, which is caused by the inevitable entry of air into ASC and the contact resistance between the electrodes during the assembly of button capacitors. According to Formula (2), the specific capacitance of ASC was calculated, as shown in Figure 7c. When the current density is 1 A g^−1^, the specific capacitance is 65.41 F g^−1^, and it maintains high rate performance. Figure 7d shows the cycling curve of ASC at 10 A g^−1^. After 8000 cycles, the capacitance retention rate is 70.6%, indicating excellent cycling performance. In addition, ACS can light up the LED after charging(Figure S4), indicating its practical application potential. The successful assembly of ASC indicates that N-GQDs have excellent practical application performance in the field of energy storage.

## 4. Conclusions

In this paper, we developed a simple hydrothermal strategy to successfully prepare N-GQDs with uniform size and systematically investigated the effects of different hydrothermal temperatures and times on the morphology and structure of N-GQDs. The abundant oxygen-containing or nitrogen-containing functional groups on the surface of N-GQDs facilitate their successful application in the field of energy storage. The results indicate that as the hydrothermal temperature or time increases, the size of quantum dots gradually increases. Excessive temperature or time can cause further growth and aggregation of N-GQDs, ultimately resulting in large-sized graphene sheets. When the hydrothermal temperature is 180 °C for 6 h, the size distribution of N-GQDs-2 is uniform, with an average size of about 3.84 nm. And the surface of N-GQDs contains abundant oxygen-containing or nitrogen-containing functional groups, which increases the surface activity of N-GQDs and accelerates the transport of electrolyte ions for electrode reactions. Additionally, N-GQDs-2 demonstrates outstanding electrochemical performance, achieving a specific capacitance of 102 F g^−1^ at a current density of 1 A g^−1^. The assembled ASC has a high specific capacitance and good cycling performance, with a capacitance retention rate of 70.6% after 8000 cycles. The successful preparation of N-GQDs broadens their application foundation in catalysis and energy storage fields.

## Data Availability

The original contributions presented in this study are included in the article/Supplementary Materials. Further inquiries can be directed to the corresponding author(s).

## Supplementary Materials

The following supporting information can be downloaded at: https://www.mdpi.com/article/10.3390/ma17246163/s1, Figure S1: AFM image of N-GQDs: (a) N-GQDs-1; (b) N-GQDs-2; (c) N-GQDs-3; (d) N-GQDs-4; (e) N-GQDs-5; (f) N-GQDs-6; Figure S2: High resolution XPS spectra of N-GQDs-2; Figure S3. The CV curve of N-GQDs-X; Figure S4. Optical photo of ASC connected LED; Table S1: Size distribution of N-GQDs.
