# Supplementary material for: Study on the Synthesis and Electrochemical Properties of Nitrogen-Doped Graphene Quantum Dots
Source: Materials (Basel). 2024 Dec 17;17(24):6163. doi: 10.3390/ma17246163 (PMC11678781; doi:10.3390/ma17246163)
Supplement: Supplementary file 1 [file materials-17-06163-s001.zip › materials-3328476-supplementary.pdf]

materials MDPI

Supplementary Materials

Study on the Synthesis and Electrochemical Properties of Nitrogen-Doped Graphene Quantum Dots

Table S1. Size distribution of N-GQDs.

	Max/nm	Min/nm	Mean/nm
**160.06**	2.95	1.48	2.13
**180.06**	4.56	2.44	3.48
**200.06**	45.90	11.56	25.89
**180.04**	3.50	1.16	1.65
**180.08**	22.49	9.05	14.19
**180.12**	36.70	9.66	20.72

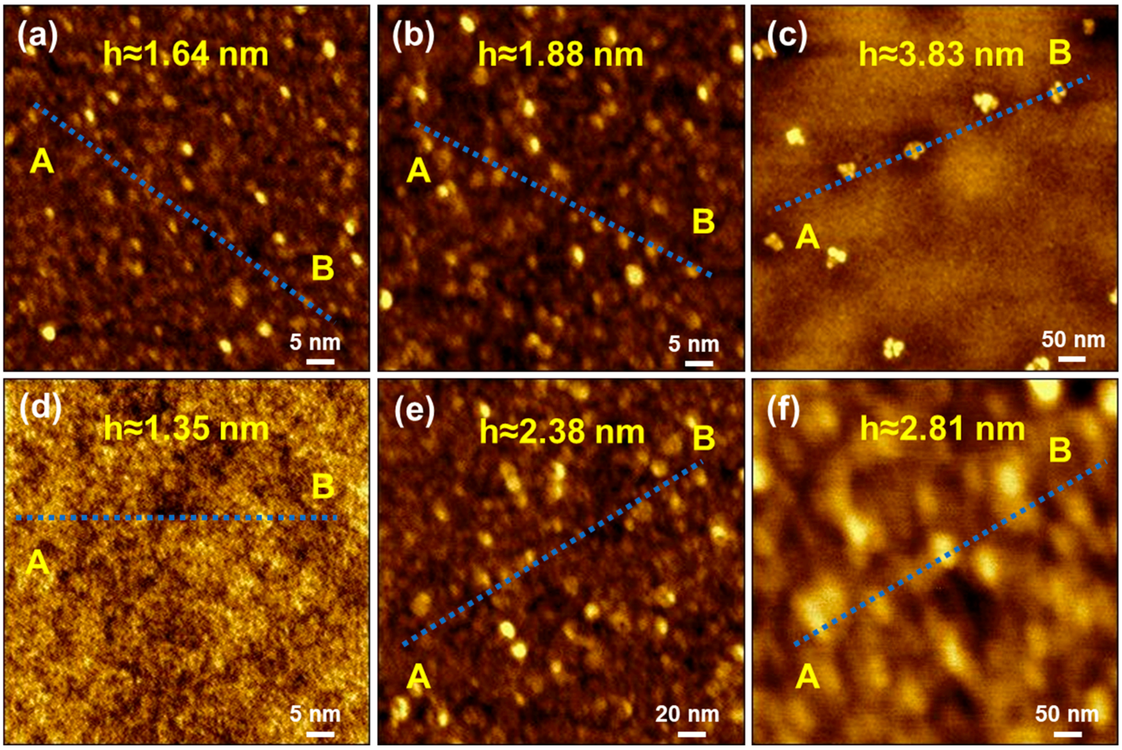

Figure S1. AFM image of N-GQDs：(a) N-GQDs-1; (b) N-GQDs-2; (c) N-GQDs-3; (d) N-GQDs-4; (e) N-GQDs-5; (f) N-GQDs-6.

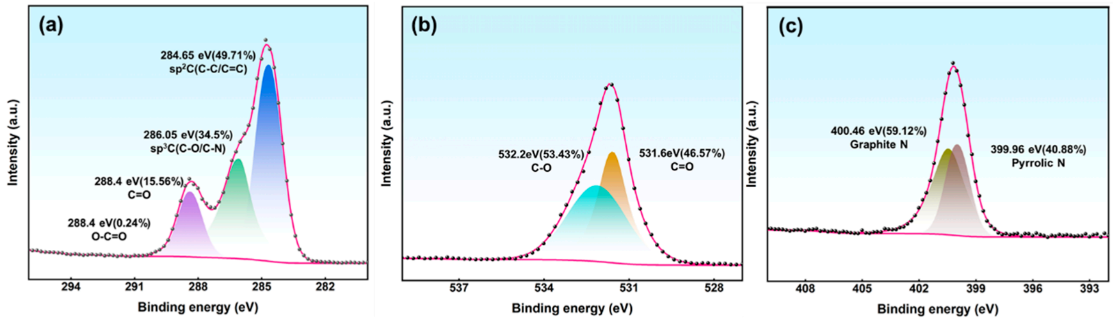

Figure S2. High resolution XPS spectra of N-GQDs-2. (a) C 1s of N-GQDs-2; (b) O 1s of N-GQDs-2; (C) N 1s of N-GQDs-2.

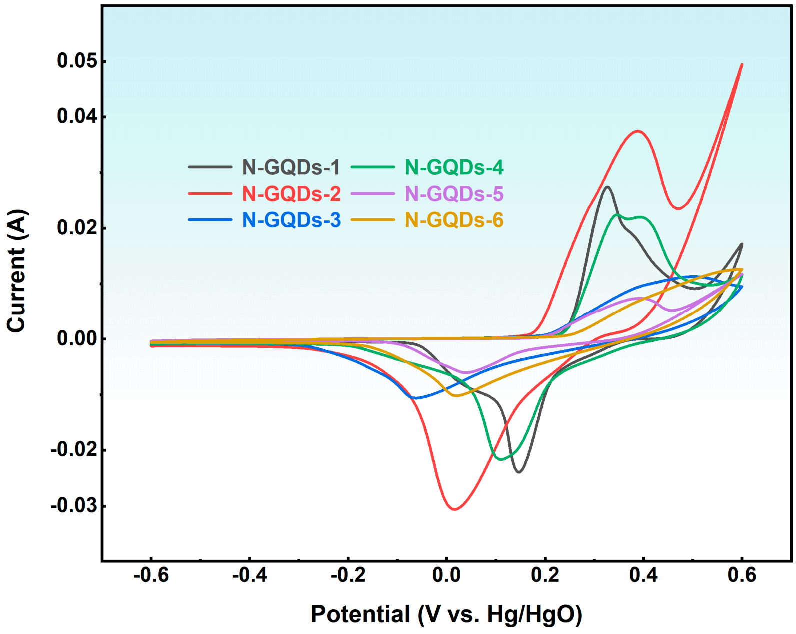

Figure S3. The CV curve of N-GQDs-X.

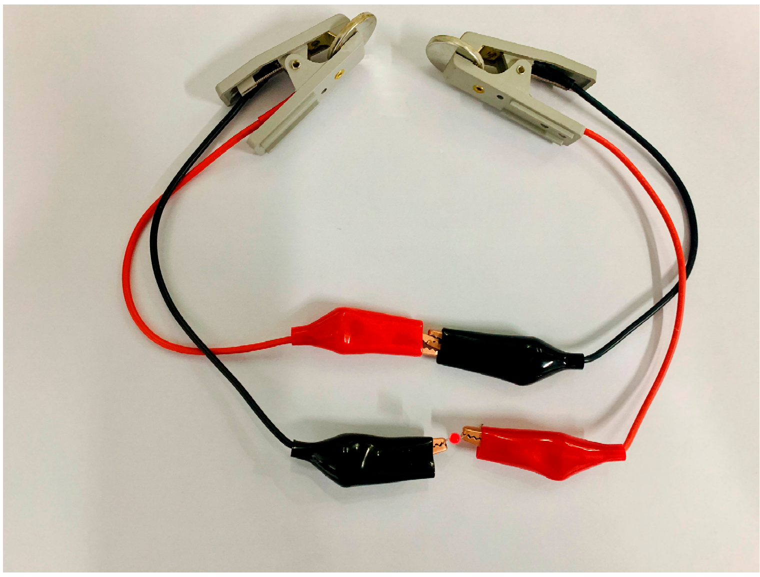

Figure S4. Optical photo of ASC connected LED.
